# Clinical spectrum and outcomes of patients with different resistance patterns of *Salmonella enterica*

**DOI:** 10.12669/pjms.38.ICON-2022.5789

**Published:** 2022-01

**Authors:** Fivzia Herekar, Samreen Sarfaraz, Muhammad Imran, Nida Ghouri, Saba Shahid, Marvi Mahesar

**Affiliations:** 1 Dr. Fivzia Herekar, FCPS, Indus Hospital and Health Network, Karachi, 75190, Pakistan; 2 Dr. Samreen Sarfaraz, FRCP, Indus Hospital and Health Network, Karachi, 75190, Pakistan; 3 Dr. Muhammad Imran, PhD, Department of Pharmacy, Iqra University, Karachi, 71500, Pakistan; 4 Nida Ghouri, M.Phil, Indus Hospital and Health Network, Karachi, 75190, Pakistan; 5 Dr. Saba Shahid, FCPS, Indus Hospital and Health Network, Karachi, 75190, Pakistan; 6 Dr. Marvi Mahesar, Indus Hospital and Health Network, Karachi, 75190, Pakistan

**Keywords:** XDR, MDR, Drug sensitive, S.*typhi*, Clinical course

## Abstract

**Background and Objective:**

Unceasing rise in cases of enteric fever, in particular extensively drug resistant (XDR) strain of Salmonella enterica, has led to a growing threat, leaving only carbapenems and azithromycin as the precious option. In this regard, we determined the burden and clinical course of XDR salmonella in comparison to multidrug-resistant (MDR) and drug sensitive (DS) strains.

**Methods:**

A retrospective chart review of 1515 Salmonella Typhi (S.typhi) culture positive patients was conducted at Indus Hospital and Health Network, Karachi from July 2017 to December 2018.

**Results:**

During our study, we observed children at the age of 5-6 years and adults at the age of 20-22 years were the chief targets of S.typhi. Further, we witnessed a rapid shift of drug resistance from MDR to XDR over the one year of study. Almost all patients presented with fever. However other signs and symptoms like malaise, body aches, anorexia, diarrhea, vomiting and abdominal pain were more common in XDR Typhoid patients. Further, the need of hospitalization, total hospital stay and mortality was also greater for XDR typhoid patients.

**Conclusion:**

There is a crucial requirement for consolidated steps to curtail the spread of XDR Salmonella tyhi disease as its management is challenging, and it is associated with increased morbidity and mortality.

## INTRODUCTION

Enteric fever is an acute systemic illness, caused by a gram negative bacterium, *Salmonella enterica*, *Serovar typhi* (*S. typhi*) or Paratyphi.^1^ The disease is characterized mainly by the presence of persistent fever and can include other symptoms such as, malaise, headache, anorexia, constipation, diarrhea and non-productive cough.^2^ Presently, enteric fever is a global health issue; annual estimated burden of disease is 11-21 million cases worldwide, resulting in 200, 000 deaths.^3^ The burden of enteric fever is the highest in the Asian region , with 93% of the global cases being reported here.^4^ In Pakistan, the incidence of enteric fever is estimated to be 413 / 100 000 person-years among children between two to four years of age and 573 / 100 000 person-years among children between 5 to 15 years of age.^5^

First line treatment for typhoid includes ampicillin, trimethoprim-sulfamethoxazole (TMP-SMZ), and chloramphenicol.^6^ In recent years, emergence of strains of *S. typhi* resistant to antibiotics commonly used for treatment has been a major concern. These multi-drug resistant (MDR) strains are no longer susceptible to orally administered first line antibiotics previously used for treatment.^7^ Moreover, resistance to fluoroquinolones, which were used to treat MDR cases, has also been reported frequently.^8^ This leaves the option of using ceftriaxone, a third-generation cephalosporin, and azithromycin, a macrolide, for treatment against these resistant strains.^6^ Rampant use of Ceftriaxone will add to the Multi drug resistant organisms including Salmonella. In MDR Salmonella, as long as it’s sensitive it is a good parenteral option with a convenient once a day dose. Moreover with clinical improvement it can be switched to oral option. Azithromycin is the only oral option left against MDR salmonella, the loss of which will mean broad spectrum carbapenems with no oral options.

A three year review of antimicrobial resistance of typhi and paratyphi in Pakistan was conducted from 2009-11; this showed resistance to fluoroquinolones increasing from 84.7% to 91.7%, along with two cases of cephalosporin resistance.^9^ In November 2016, an outbreak of ceftriaxone-resistant *S. typhi* was detected in Hyderabad, Sindh,^10^ later spreading to Karachi. This was the largest outbreak of ceftriaxone-resistant *S. typhi* that has been reported globally.^8^ This leaves very limited options for the treatment of new cases of enteric fever; carbapenems, azithromycin and tigecycline. Unfortunately, cases of azithromycin resistance are now being reported in South-east Asia as well.^11^

Given the burden of disease, which may be underreported due to lack of proper facilities of microbiological diagnosis, case fatality rate of enteric fever is expected to be higher and further rise. The clinical paradigms of patients diagnosed with XDR strain of enteric fever are slowly surfacing, with greater complications, and protracted clinical course and mortalities. With the paucity of appropriate diagnostic tools and limited antibiotics, clinicians will soon be at a loss on how to successfully treat this disease.

This study was conducted to determine the clinical course of XDR salmonella, comparing the severity of the strain to the MDR and drug-sensitive strains in both adult and pediatric population in a tertiary care setting. The study further evaluates the burden of the XDR salmonella strain in all cases of enteric fever, along with the complications, treatment course and clinical outcomes.

## METHODS

A retrospective chart review was conducted of patients diagnosed with enteric fever at Indus Hospital and Health Network from 1st July, 2017 to 31st December, 2018. All patients, both adult and pediatric, with culture-proven enteric fever were included in the study. The data was extracted through the Health Management Informatics System (HMIS). IRB approval was obtained and the approval number is: IRD_IRB_2018_08_009 on 29^th^ August 2018.

Blood cultures were performed using 5 ml of blood, drawn under aseptic measures, and collected in BacT/Aert culture bottles, which were then sent to the microbiology laboratory for analysis. Antibiotic susceptibility for antibiotics was tested using Disc Diffusion Method. Clinical and Laboratory Standards Institute (CLSI) guideline was followed to interpret the susceptibility pattern.

A pre-designed questionnaire was used to record detailed information about patient demographics, signs and symptoms, clinical course, treatment, complications, and final outcomes. Laboratory parameters were also recorded to determine the severity and course of the disease; these included complete blood count (CBC), liver function test (LFTs), serum electrolytes, Urea and Creatinine.

## RESULTS

**Fig.1:**
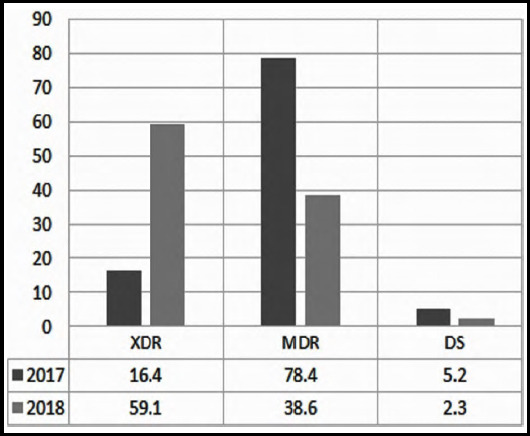
Yearly distribution of Drug resistance typhoid.

The study enrolled 1515 culture-proven enteric fever patients (Male: 890; Female: 625) (Table-I). The ratio of children was much higher than adults (88 % children up to 15 years age). The age groups most affected by *S.typhi* infection in our study include children 5-6 year age and adults 20-22 year age. (Table-I). In terms of drug resistance, we have observed three different strains of Salmonella with alarming numbers of resistant strains i.e. 50.5 % of XDR, 46.6 % of MDR and only 2.9 % of drug sensitive strains (Table-I). The patient inflow was much higher in the period of May to November, more specifically in July, which represented the peak season of bacterial illness. Over the one year, there was a rapid shift of predominant *S.typhi* strain from MDR to XDR i.e. 78.4% MDR patients were registered in 2017 as compared to 38.6% in 2018, while XDR cases increased from 16.4% in 2017 to 59.1% in 2018 leading to a further decline in drug sensitive cases from 5.2% to 2.3% respectively (Fig.1).

**Table I:** Characteristics of enrolled patients.

Category	Characteristics	XDR		MDR		Drug sensitive	

		Adult	Paeds	Adult	Paeds	Adult	Paeds
Demographics	Age, Median, Years	22	5	21	6	20	5
	Male	51	391	50	374	10	14
	Female	35	288	27	255	3	17
Severity	Fever, Median (Range), Days	15 (1-90)	7.5 (1-150)	14.5 (1-180)	7 (1-210)	15 (3-90)	7 (1-90)
	Hospitalization, Median (Range), Days	8 (2-20)	6 (1-19)	5 (2-14)	4 (1-14)	4 (2-6)	4.5 (2-11)
Therapy	Single drug	30	245	31	307	7	11
	Multiple Drug	23	201	13	87	1	7
Outcomes	Known (Cured-Death)	40 (40-0)	270 (266-4)	29 (29-0)	162 (161-1)	2 (1-1)	13 (13-0)
	Lost to follow or LAMA	37	358	44	454	11	18
	Referred due to unavailability of bed	9	51	4	13	0	0

**Fig.2:**
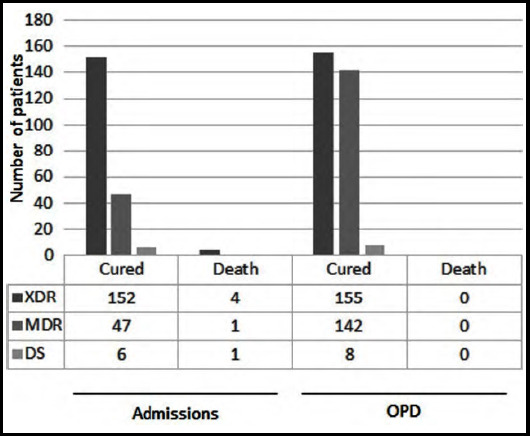
Known outcomes of enrolled patients.

The total number of patients who needed admission was 288 (19 %), while remaining patients were treated in outpatient (OPD) or daycare departments. Amongst patients requiring hospitalization, 211 were admitted at the hospital, whereas 77 were referred due to unavailability of space. A huge number of patients (922 patients, 60.85 %) were lost to follow or left against medical advice during our study, hence their outcomes were unknown. Almost all patients (admissions & OPD) presented with fever along with other sign and symptoms. Among adults and children, fever lasted for approximately fifteen days and seven days respectively (Range: 1-210 days) with no significant difference between drug sensitive and resistant *S.typhi* species. Other frequently noticed symptoms were malaise, body aches, anorexia, diarrhea, vomiting and abdominal pain. During the study, it was observed that patients with above symptoms were most likely to have XDR typhoid. Taking malaise, body aches and diarrhea as reference, 23.66%, 10.84% and 26.53% of XDR typhoid patients reported these symptoms while only 3.54%, 8.25% and 19.54% of MDR typhoid patients and 6.81%, 9.09% and 9.09% of DS typhoid patients reported these symptoms respectively. In addition, the severity of disease was portrayed by days of hospitalization and mortality. The need for hospitalization was greater among XDR patients in comparison to MDR and DS. Out of 211 hospitalized patients, 156 (73.93 %) were diagnosed as XDR while 48 were MDR and seven belonged to DS (Fig.2). Furthermore, XDR patients were hospitalized for longer duration (eight days for adults and six days for children) than MDR and DS (4-5 days in both children and adults). In addition, mortality rate was also higher among XDR typhoid patients compared to those with MDR and DS typhoid. (Four, one and one respectively). One possible reason for increased hospitalization need and higher mortality in XDR patients was the complication rate. Abdominal pain and jaundice were the common manifestations of infection, followed by CNS manifestations, PR bleeding, cholecystitis and other (Table-II).

**Table II:** Commonly reported complications in enrolled patients.

Complication	XDR	MDR	DS
Jaundice	24	9	2
Acute abdomen	83	27	1
PR bleeding	9	5	1
Metastatic abscess	2	1	0
CNS manifestations	13	3	0
Cholecystitis	6	0	1
Septic arthritis	2	0	0
Death	4	1	1

With lowered antibiotic sensitivity and minimal drug options for XDR salmonella, treatment has been very challenging. In our study, either azithromycin or meropenem was used as monotherapy or in combination in XDR patients while cephalosporins were used in MDR patients. In total, monotherapy was given more frequently (65.52% patients) in comparison to a combination of drugs. However, considering only cured patients, combination therapy was preferred over monotherapy in XDR patients (62.8%) while vice versa in MDR patients (40.9%).

## DISCUSSION

This study witnessed a rapid shift of drug resistance from MDR to XDR over the one year of study. Further, we observed that the need of hospitalization, total hospital stay and mortality was greater for XDR typhoid patients. The disease burden of Enteric fever is highest in Asia and Africa. Although the environmental drivers for the seasonality of the disease are not well understood, however it has been assumed that it is related to monsoon and flooding in low income countries with poor infrastructure. The peak season for typhoid was seen in July in our study, while the numbers remained high from May through November, this correlated well with the seasonal pattern of the disease documented for the region of Asia.^12^ In 2016, an extremely resistant strain of salmonella was documented for the first time in Pakistan.^9^ This was depicted in our study with the gradual shift of resistance pattern from MDR to XDR over one year eventually accounting for more than 50% of the cases of *Salmonella typhi*.

Conventionally, enteric fever has been a mild disease treated in the outpatient department, but now with increasing antibiotic resistance, the need for hospitalization has increased due to prolonged, severe disease, complications and the need for parenteral antibiotics. In a prospective surveillance for enteric fever done in three Asian countries, Pakistan, Bangladesh and Nepal, 32% patients were hospitalized.^13^ The hospitalization rate in our study was 13.92% (excluding the 5% additional patients who were unable to get inpatient accommodation due to space constraints). Of these 73.93% patients had XDR salmonella infection. The growing resistance to antibiotics has shown adverse implications in enteric fever. In a systematic review evaluating clinical characteristics of enteric patients, those with MDR salmonella had delayed presentation to the hospital, with a more complicated course compared to the drug sensitive arm.^14^ Complications are noted in 10-15 % of hospitalized patients, the most commonly reported being GI bleed, intestinal perforation, altered mental status, arthritis and jaundice.^15^ Similarly in our study, duration of hospitalization, was prolonged in the XDR salmonella patients leading to higher number of complications subsequently, compared to MDR and DS. The reason for admission in 10% of the patients was some sort of complication while the remainder required parenteral antibiotics. The most common complications in our patients in chronological order were acute abdomen, jaundice, CNS manifestations, PR bleeding, cholecystitis, metastatic abscesses and septic arthritis. Seeding of salmonella was seen in the form of tuboovarian abscess, gallbladder empyema and abscess in the elbow in our study. This tendency of salmonella to seed in other locations has also been documented in other case reports.^16^-^18^ Most of these complications in our patient pool however were more common in patients with XDR salmonella. Salmonella is known to be commoner is pediatric age group. Children tend to exhibit fewer symptoms as may not be able to express, yet when they do manifest symptoms they are shorter and sicker compared to adults who tolerate longer and seek medical advice a little delayed compared to pediatric age group, as observed in our study. Treatment has been very challenging with increasing resistance and no definitive guidelines, especially for the relatively new XDR salmonella. There has been strong evidence of activity of azithromycin^19^ against MDR salmonella however not much evidence for the use of carbapenems. Carbapanems are used as last resort antibiotics for multidrug resistance gram negative bacteria. In a study evaluating treatment strategies in XDR salmonella patients, in Pakistan during the period of 2017-2018, azithromycin and meropenem monotherapy was given in 27% and 25% patient respectively, while 48% received a combination of both.^20^ There was similar time to fever defervescence in all three groups, however the cost in the azithromycin group was substantially lower. In our study, 37.2% patients of XDR with known outcomes were cured on monotherapy i.e. either azithromycin or meropenem, while 62.8% (n=182) were cured with combination drug therapy. In MDR 59.1% (n=91) were cured on monotherapy, while 40.9% (n=63) were cured on multidrug therapy. Monotherapy in MDR was mainly cephalosporins.

The case fatality rate previously concurred was 1%,^4^ however in a recent meta-analysis, done in 2018, it was estimated to be 2.45% overall and 4.45% for those hospitalized.^21^ In this meta-analysis, there was considerable heterogeneity in CFR between studies, which could not be explained despite evaluating for World bank income level, different serovars, HIV status etc. In fact, this heterogeneity was even seen in studies from the same country, In our study, six hospitalized patients expired. Four of these being of pediatric group infected with XDR salmonella. This is in concordance with the known fact that children are less immune-competent as compared to adults and may have more complicated bacterial infections. The case fatality rate for our hospitalized patients was hence 0.4%. However, as a major proportion of the patients were loss to follow or referred due to non-availability of beds, the overall mortality may be an underestimate. These observations are of major concern especially in low income countries with a lack of robust health care system.

## CONCLUSION

Growing antibiotic resistance in *Salmonella enterica* has made it a complicated disease to treat. This may be of grave consequence especially in low middle income countries where access to health care facilities and expensive antibiotics is not available to many. Steps should be taken to improve antibiotic prescribing practices and provide unified guidelines for management of extremely drug resistant *Salmonella enterica*.

### Authors Contribution:

**FH, SS & SS:** Conceived, designed and supervised the study.

**FH & MI:** Did manuscript writing & editing.

**MI, NG & MM:** Did data collection & statistical analysis.

**FH:** Did review, analysis and final approval of manuscript.
